# Review on skeletal disorders caused by *Staphylococcus* spp. in poultry

**DOI:** 10.1080/01652176.2022.2033880

**Published:** 2022-02-08

**Authors:** Gustaw M. Szafraniec, Piotr Szeleszczuk, Beata Dolka

**Affiliations:** Department of Pathology and Veterinary Diagnostics, Institute of Veterinary Medicine, Warsaw University of Life Sciences – SGGW, Warsaw, Poland

**Keywords:** Poultry, chicken, welfare, Staphylococcus, infectious disease, bacterial chondronecrosis, femoral head necrosis, lameness, skeletal disorders

## Abstract

Lameness or leg weakness is the main cause of poor poultry welfare and serious economic losses in meat-type poultry production worldwide. Disorders related to the legs are often associated with multifactorial aetiology which makes diagnosis and proper treatment difficult. Among the infectious agents, bacteria of genus *Staphylococcus* are one of the most common causes of bone infections in poultry and are some of the oldest bacterial infections described in poultry. Staphylococci readily infect bones and joints and are associated with bacterial chondronecrosis with osteomyelitis (BCO), spondylitis, arthritis, tendinitis, tenosynovitis, osteomyelitis, turkey osteomyelitis complex (TOC), bumblefoot, dyschondroplasia with osteomyelitis and amyloid arthropathy. Overall, 61 staphylococcal species have been described so far, and 56% of them (34/61) have been isolated from clinical cases in poultry. Although *Staphylococcus aureus* is the principal cause of poultry staphylococcosis, other *Staphylococcus* species, such as *S. agnetis, S. cohnii*, *S. epidermidis, S. hyicus, S. simulans*, have also been isolated from skeletal lesions. Antimicrobial treatment of staphylococcosis is usually ineffective due to the location and type of lesion, as well as the possible occurrence of multidrug-resistant strains. Increasing demand for antibiotic-free farming has contributed to the use of alternatives to antibiotics. Other prevention methods, such as better management strategies, early feed restriction or use of slow growing broilers should be implemented to avoid rapid growth rate, which is associated with locomotor problems. This review aims to summarise and address current knowledge on skeletal disorders associated with *Staphylococcus* spp. infection in poultry.

## 1. Introduction

Skeletal disorders that lead to poor leg health and diminished locomotory function (lameness or leg weakness) are one of the most common and important issues in modern meat-type poultry production (McNamee and Smyth 2000; Wideman 2016). Lameness in poultry translates into poor welfare and significant economic losses due to elevated feed conversion ratio (FCR), low production results, increased mortality, and culling. Many of the causes of lameness are associated with pain and birds with critically impaired motility are unable to reach drinkers and feeders. Poor welfare has a negative effect on the health and production parameters of birds and affects the quality of animal products (Kestin et al. 1992; Weeks et al. 2000; Bradshaw et al. 2002; Gocsik et al. 2017; Granquist et al. 2019). Annual economic losses due to leg problems in the USA in early 1990s were estimated at $80–120 million in the broiler industry and $32–40 million in the turkey industry (Sullivan 1994). In the UK, BCO was responsible for losses of 0.75% of all broiler placements, which at the time cost the UK broiler industry £3 million annually (McNamee and Smyth 2000). By another estimation, BCO contributed to 0.5–0.7% of losses by mortality and culling which represented 3.75 million birds and cost the UK broiler industry £4.7 million annually (Butterworth 1999). It has been estimated that each year, 12.5 billion broiler chickens worldwide experience leg problems (Nicol 2013). According to the European Commission (DG SANTE. 2016), around 30% of all broiler chickens reared in intensive production systems exhibit some form of legs disorder. The severity of lameness is usually gauged by gait scoring, e.g., as proposed by Kestin et al. (1992), where chickens are observed, and points are granted on a six-point scale, where 0 equals to normal gait and 5 equals to complete immobility. The incidence of lameness (defined as gait score ≥3) in broiler chicken flocks have been estimated at 14.5% in Sweden (Gocsik et al. 2017), 15.6% in 4 European countries (France, Italy, the United Kingdom, the Netherlands) (Bassler et al. 2013), 19% (Granquist et al. 2019) and 24.6% (Kittelsen et al. 2017) in Norway, 26% (Kestin et al. 1992) and 27.6% (Kestin et al. 1992) in the United Kingdom, 30.1% (gait score ≥2) in Denmark (Sanotra et al. 2001), and 57% in various European countries (the Netherlands, Italy, Belgium, United Kingdom) (de Jong et al. 2011).

The problem of lameness in poultry has a complex aetiology that includes genetic, environmental, nutritional, and infectious diseases. Fast-growing broilers experience lameness most often, as their immature skeletal systems cannot withhold the rapidly increasing body mass (Thorp and Waddington 1997; Wideman and Prisby 2012; Prisby et al. 2014). Among infectious factors, staphylococci have been recognized as one of the most important bacterial pathogens responsible for skeletal infections in poultry. Besides staphylococci, other bacterial species such as *Enterococcus* spp. and *Escherichia coli* are often isolated from bone lesions (Huff et al. 2000; Dinev 2009; Wideman 2016; Wijesurendra et al. 2017).

The members of the genus *Staphylococcus* (Rosenbach 1884) are saprophytic bacteria that colonise the surface of the skin and mucous membranes of humans and animals, including poultry (Cooper and Needham 1976; Nagase et al. 2002; Syed et al. 2020). Staphylococci are found in animal products as well as in the environment – in dirt, dust, air, and water. They belong to opportunistic pathogens and important nosocomial pathogens. Furthermore, staphylococcal enterotoxins produced by enterotoxigenic strains in food cause staphylococcal food poisoning which belongs to the most common food-borne diseases in the world (Pinchuk et al. 2010; Hennekinne et al. 2012; Sergelidis and Angelidis 2017; Algammal et al. 2020).

Staphylococci are ubiquitous in poultry farms and hatcheries. They can be responsible for the contamination of poultry feed, egg incubators and hatchers (Devriese 1980; Thompson et al. 1980; Devriese 1990; McCullagh et al. 1998; Rodgers et al. 2003; Parveen et al. 2017). The prevalence of *Staphylococcus* spp. and *S. aureus* on the eggshell surface of chicken table eggs was 20.45% and 10.45%, respectively (Pondit et al. 2018). Eggshell microbiota is a mix of faecal microbiota of broiler breeder that laid the egg and environmental microbiota (Maki et al. 2020; Trudeau et al. 2020). *Staphylococcaceae* may form 1.09% of eggshell microbiota. Recent data suggest that eggshell microbiota, feed or drinking water do not play a major role in the intestinal colonisation of newly hatched chicks (Volf et al. 2021).

Staphylococci are common components of microbiota of poultry houses, found in litter, dust, drinking water, air, and on farm equipment. There are no studies comparing the diversity of poultry house staphylococci and staphylococci isolated from skeletal disorders. One study in Northern Ireland showed that the personnel of a hatchery and broiler breeder farms can carry on their hands biotypes of *S. aureus* associated with skeletal disease in poultry, thus possibly contributing to the dissemination of this pathogen (Rodgers et al. 1999). The genus *Staphylococcus* was present in significant amounts throughout the rearing period in all litter samples collected from five turkey farms in Arkansas, USA. It was the second most abundant genus out of four, preceded only by *Corynebacterium* (Adhikari et al. 2020). Brooks et al. (2010) estimated staphylococci to account for approximately 90% of all bacteria cultured from litter samples on a broiler farm in Mississippi, USA.

Bacteria in poultry house bioaerosol may come from soil, feed, water, litter, and birds themselves (Lonc and Plewa 2009). Coagulase-negative staphylococci seem to be much more often isolated from poultry farm samples than coagulase-positive staphylococci. A study of microbial air contamination in three breeding houses in Poland showed that coagulase-negative staphylococci were the most abundant bacteria isolated from air samples, constituting nearly 42% of all isolated microorganisms (Bródka et al. 2012). Similarly, Sanz et al. (2021) found mostly coagulase-negative staphylococci in litter and air samples (both from the inside and the outside of a farm) of a broiler farm in Spain, with *S. saprophyticus* being the most abundant staphylococcus species (40.7%). Oppliger et al. (2008) isolated *S. xylosus* and *S. equorum* from air samples and demonstrated that bacterial load in air samples increased throughout the rearing period of chickens. *Staphylococcus cohnii* was the most often isolated bacterium from air samples in Texas (Nonnenmann et al. 2010). Just et al. (2011) found staphylococci to be the most common bacteria isolated from air samples, and the concentration of bacteria was much higher in floor housing systems than in cage housing systems. In Germany, methicillin-resistant *S. aureus* was found in most (77.8%) air samples in the broiler and turkey houses tested (Friese et al. 2013).

In poultry, staphylococcosis is a term covering several clinical syndromes caused by *Staphylococcus* spp., in which the aetiological agent, after penetrating the damaged skin or mucous membrane, infiltrates the deeper located tissues. Disease usually occurs when the natural immunity of the host is impaired (Harry 1967; Huff et al. 2000; McNamee and Smyth 2000; Wideman and Pevzner 2012). Staphylococcal infections are some of the oldest bacterial infections described in poultry. The earliest report (1870) comes from the case of lameness in young geese in Prussia (Hinshaw and McNeil 1952).

Coagulase-positive staphylococci, including *S. aureus*, are considered to be the biggest health concern in poultry. Infections caused by other *Staphylococcus* species, including coagulase-negative staphylococci, have been reported less frequently (Rich 2005; Peton and Le Loir 2014; Pyzik et al. 2019; González-Martín et al. 2020). However, in a study by Wijesurendra et al. (2017), a total of 41 staphylococcal isolates were recovered from broiler bones with histopathological signs of inflammation, and only six of these isolates (15%) were categorised as coagulase-positive *Staphylococcus* spp. Staphylococci showed varied prevalence in poultry flocks depending on geographic location. In previous years, the frequency of *Staphylococcus* spp. infections in chickens, turkeys, waterfowl was determined at 10.5% (60/572), and most isolates (71.7%; 43/60) came from chickens. Staphylococci were isolated from tissue lesions (organs, joints) (Wieliczko et al. 2002). In another study, the frequency of *Staphylococcus* spp. infections in various poultry species and production purposes was determined at 10.8% (Marek et al. 2016). Another readily available report that focused solely on *S. aureus* showed that the rate of asymptomatic infections with *S. aureus* may reach 57% of poultry flocks in Algeria. The highest flock prevalence of *S. aureus* (nasal carriage) was found in turkeys (75.6%), in breeding hens (52.8%), in laying hens (48.8%), in broilers (48.4%) (Benrabia et al. 2020). The prevalence of MRSA turkey flocks (cloacal and tracheal swabs) in Germany can be as high as 90%. Furthermore, all MRSA-positive flocks also had their corresponding dust samples positive for MRSA (Richter et al. 2012).

Overall, 61 staphylococcal species have been described so far (correct names and validly published under the International Code of Nomenclature of Prokaryotes – ICNP) (Parte et al. 2020). Table 1 shows all *Staphylococcus* species found in poultry (n** **=** **35), and 56% (34/61) of them were isolated from clinical cases in poultry.

**Table 1.** Alphabetical list of *Staphylococcus* species found in poultry (n** **=** **35). All but one were isolated from clinical cases in poultry (except *S. caprae*).

*Staphylococcus* spp.	References
*S. agnetis*	(Al-Rubaye et al. 2015; Poulsen et al. 2017; Thøfner et al. 2019; Szafraniec et al. 2020; Ekesi et al. 2021)
*S. arlettae*	(Awan and Matsumoto 1998; Wieliczko et al. 2002; Pyzik et al. 2019)
*S. aureus*	(Munger and Kelly 1973; Kibenge et al. 1983; Bayyari et al. 1994; Awan and Matsumoto 1998; McNamee and Smyth 2000; Wieliczko et al. 2002; Al-Rubaye et al. 2015; Tsai et al. 2015; Braga et al. 2016; Marek et al. 2016; Wijesurendra et al. 2017; Gornatti-Churria et al. 2018; Heidemann Olsen et al. 2018; Pyzik et al. 2019; Thøfner et al. 2019; Ekesi et al. 2021)
*S. auricularis*	(Awan and Matsumoto 1998)
*S. capitis*	(Awan and Matsumoto 1998; Marek et al. 2016; Pyzik et al. 2019)
*S. caprae*	(Syed et al. 2020)
*S. carnosus*	(Awan and Matsumoto 1998)
*S. caseolyticus*	(Awan and Matsumoto 1998)
*S. chromogenes*	(Marek et al. 2016; Pyzik et al. 2019)
*S. cohnii*	(Scanlan and Hargis 1989; Wieliczko et al. 2002; Marek et al. 2016; Pyzik et al. 2019; Ekesi et al. 2021)
*S. condimenti*	(Marek et al. 2016)
*S. epidermidis*	(Scanlan and Hargis 1989; Awan and Matsumoto 1998; Al-Rubaye et al. 2015; Marek et al. 2016; Gornatti-Churria et al. 2018; Pyzik et al. 2019; Ekesi et al. 2021)
*S. equorum*	(Marek et al. 2016; Pyzik et al. 2019)
*S. felis*	(Marek et al. 2016; Pyzik et al. 2019)
*S. gallinarum*	(Awan and Matsumoto 1998)
*S. haemolyticus*	(Bayyari et al. 1994; Wieliczko et al. 2002; Marek et al. 2016; Pyzik et al. 2019)
*S. hominis*	(Awan and Matsumoto 1998; Al-Rubaye et al. 2015; Marek et al. 2016; Pyzik et al. 2019)
*S. hyicus*	(Cheville et al. 1988; Tate et al. 1993; McNamee and Smyth 2000; Wieliczko et al. 2002; Chénier and Lallier 2012; Marek et al. 2016; Heidemann Olsen et al. 2018)
*S. intermedius*	(Scanlan and Hargis 1989; Awan and Matsumoto 1998; Pyzik et al. 2019)
*S. kloosii*	(Awan and Matsumoto 1998)
*S. lentus*	(Scanlan and Hargis 1989; Bayyari et al. 1994; Awan and Matsumoto 1998; Wieliczko et al. 2002; Marek et al. 2016; Pyzik et al. 2019; Thøfner et al. 2019)
*S. lugdunensis*	(Marek et al. 2016)
*S. pasteuri*	(Pyzik et al. 2019)
*S. pettenkoferi*	(Marek et al. 2016)
*S. pisifermentans*	(Awan and Matsumoto 1998)
*S. pseudintermedius*	(Pyzik et al. 2019)
*S. saprophyticus*	(Wieliczko et al. 2002; Al-Rubaye et al. 2015; Marek et al. 2016; Pyzik et al. 2019)
*S. schleiferi*	(Marek et al. 2016; Pyzik et al. 2019)
*S. sciuri*	(Scanlan and Hargis 1989; Wieliczko et al. 2002; Marek et al. 2016; Pyzik et al. 2019)
*S. simiae*	(Marek et al. 2016)
*S. simulans*	(Scanlan and Hargis 1989; Awan and Matsumoto 1998; McNamee and Smyth 2000; Wieliczko et al. 2002; Marek et al. 2016; Stępień-Pyśniak et al. 2017; Pyzik et al. 2019; Thøfner et al. 2019; Ekesi et al. 2021)
*S. ureilyticus* (homotypic synonyms: *S. cohnii* subsp. *urealyticus, S. cohnii* subsp. *urealyticum*)	(Awan and Matsumoto 1998; Tsai et al. 2015)
*S. vitulinus*	(Marek et al. 2016; Pyzik et al. 2019)
*S. warneri*	(Scanlan and Hargis 1989; Marek et al. 2016; Pyzik et al. 2019)
*S. xylosus*	(Awan and Matsumoto 1998; McNamee and Smyth 2000; Wieliczko et al. 2002; Al-Rubaye et al. 2015; Marek et al. 2016; Gornatti-Churria et al. 2018; Pyzik et al. 2019)

In poultry, staphylococci are usually responsible for local and chronic infections that rarely take the form of systemic infections (Andreasen 2020). Staphylococci have evolved mechanisms to survive phagocytosis by mononuclear cells and heterophils, so that they may persist in the host and form a reservoir for recurrent infections (Thwaites and Gant 2011). Staphylococci were isolated from dead chicken embryos as well as from one day old chicks and adult birds (Sahu and Munro 1969; Kibenge et al. 1983; Orajaka and Mohan 1985; Brash et al. 2013; Amer et al. 2017; Andreasen 2020). A high incidence of *Staphylococcus* isolation from dead-in-shell chicken embryos (in 7.6% showing developmental abnormalities) may confirm the contribution of staphylococcal infections to the embryonic mortality and reduced hatchability (Hananeh et al. 2021).

Pathological conditions associated with staphylococci in poultry are shown in Table 2, and these concerning the skeleton have been highlighted and discussed. However, it should be noted that these disorders could also be caused by other bacteria. Although different *Staphylococcus* species may be involved in various pathological conditions (Marek et al. 2016; Andreasen 2020; Szafraniec et al. 2020), some of them have been more often associated with specific infection, e.g., *S. aureus* with bumblefoot, gangrenous dermatitis (Huff et al. 2000; Wieliczko et al. 2002; Heidemann Olsen et al. 2018; Andreasen 2020); *S. hyicus* with turkey stifle joint osteomyelitis, eye infection (blepharitis, conjunctivitis), acantholytic dermatitis (Cheville et al. 1988; Tate et al. 1993; Chénier and Lallier 2012); *S. simulans* and *S. agnetis* with endocarditis (Poulsen et al. 2017; Stępień-Pyśniak et al. 2017). A broad range of *Staphylococcus* species have been isolated from arthritis, and *S. aureus* was usually the most commonly identified (83.3% 15/18; 58.3% 14/24) (Wieliczko et al. 2002; Tsai et al. 2015). Other staphylococci cultured from septic joints and bone lesions included *S. agnetis, S. capitis, S. caseolyticus, S. epidermidis, S. gallinarum, S. haemolyticus, S. hominis, S. intermedius, S. lentus, S. simulans, S. ureilyticus, S. xylosus* (Bayyari et al. 1994; Awan and Matsumoto 1998; Wieliczko et al. 2002; Al-Rubaye et al. 2015; Tsai et al. 2015; Ekesi et al. 2021). Although *E. coli* are the most prevalent bacteria causing infections in chicks, *S. aureus*, *S. epidermidis*, *S. haemolyticus*, *S. scuiri*, *S. xylosus* have also been involved in omphalitis and first-week mortality (Reda et al. 2013; Amer et al. 2017).

**Table 2.** *Staphylococcus*-associated infections in poultry. Grey shaded areas show infections of the skeletal system.

*Staphylococcus*-associated infections in poultry	References
Bacterial chondronecrosis with osteomyelitis (BCO)	(McNamee and Smyth 2000; Al-Rubaye et al. 2015; Jiang et al. 2015; Wijesurendra et al. 2017)
Arthritis	(Devriese 1990; Awan and Matsumoto 1998; Wieliczko et al. 2002; Brash et al. 2013; Tsai et al. 2015; Marek et al. 2016; Andreasen 2020)
Tendinitis, tenosynovitis	(Hinshaw and McNeil 1952; Nairn 1973; Kibenge et al. 1983; Andreasen et al. 1993; Brash et al. 2013; Andreasen 2020)
Osteomyelitis	(Nairn and Watson 1972; Nairn 1973; Andreasen et al. 1993; Tate et al. 1993; Brash et al. 2013; Andreasen 2020)
Spondylitis(vertebral osteomyelitis)	(Devriese 1990; Brash et al. 2013; Braga et al. 2016)
Bumblefoot (ulcerative pododermatitis, footpad dermatitis)	(Sahu and Munro 1969; Hester 1994; Wilcox et al. 2009; Heidemann Olsen et al. 2018; Youssef et al. 2019)
Dyschondroplasia with osteomyelitis	(Wyers et al. 1991; Rath et al. 1994; Huff et al. 2000)
Amyloid arthropathy	(Landman et al. 1998; Landman 1999)
Turkey osteomyelitis complex (TOC) previously synovitis, osteomyelitis and green liver syndrome (SOG) = green liver-osteomyelitis complex in turkey	(Bayyari et al. 1994; Brash et al. 2013; Andreasen 2020)
Omphalitis and yolk sacculitis	(Brash et al. 2013; Marek et al. 2016; Amer et al. 2017)
Gangrenous dermatitis	(Brash et al. 2013; Gornatti-Churria et al. 2018; Andreasen 2020)
Cellulitis	(Brash et al. 2013; Marek et al. 2016)
Scabby hip syndrome	(Scanlan and Hargis 1989; Brash et al. 2013)
Subdermal fibriscess	(Brash et al. 2013)
Acantholytic folliculitis and epidermitis	(Chénier and Lallier 2012)
Comb necrosis	(Nakamura et al. 1997)
Enteritis	(Sahu and Munro 1969)
Pneumonia	(Linares and Wigle 2001; Igbokwe et al. 2012)
Airsacculitis	(Sahu and Munro 1969)
Salpingitis, salpingoperitonitis	(Jordan et al. 2005)
Peritonitis	(Sahu and Munro 1969)
Conjunctivitis	(Cheville et al. 1988; Andreasen 2020)
Blepharitis	(Cheville et al. 1988)
Endocarditis	(Brash et al. 2013; Poulsen et al. 2017; Stępień-Pyśniak et al. 2017)
Granulomas (in liver, lungs)	(Munger and Kelly 1973; Linares and Wigle 2001)
Septicaemia	(Sahu and Munro 1969; Devriese 1990; Brash et al. 2013; Poulsen et al. 2017; Andreasen 2020)

## 2. Virulence factors

Staphylococci have a wide range of virulence factors (Wright and Nair 2010). Here we present only a few selected factors that may play a role in skeletal infections. It should be noted that the vast majority of studies of staphylococcal virulence factors in skeletal disorders have been conducted on mammalian models.

An important factor enabling staphylococci to colonise bones and joints is their ability to adhere to the components of the extracellular matrix of host cells. Adhesion to the host matrix, ​​the first step of successful bacterial infection, is enabled by proteins called adhesins. Adhesins can divided into microbial surface components recognizing adhesive matrix molecules (MSCRAMMs), which are bound to the cell wall (Foster and Höök 1998), and secretable expanded repertoire adhesive molecules (SERAMs), which are secreted extracellularly (Chavakis et al. 2005). In addition to mere adhesion of bacteria to the extracellular matrix, some adhesins are able to manipulate the host’s immune response and also allow for the internalisation of bacterial cells (Clarke and Foster 2006).

Internalisation is associated with the occurrence of forms of staphylococci known as small colony variants (SCVs). They are naturally occurring subpopulations of bacteria with distinctive phenotypic traits (Proctor et al. 2006). Staphylococcal SCV phenotypes are characterised by decreased induction of immune response in the host, decreased expression of exotoxins, and increased expression of adhesins. It is the latter that allows SCVs to penetrate inside host cells and remain dormant there (Proctor et al. 2006). The emergence of SCVs is probably related to the adaptation of bacterial cells to an unfavourable environment, determined by factors such as low pH (Leimer et al. 2016; Perez and Patel 2017), low temperatures, the presence of antibiotics (Onyango et al. 2013), long-term lack of nutrients (Bui et al. 2015), and oxidative stress (Lee et al. 2020). After the adverse factors have ceased, SCVs may revert to their original phenotype, which may result in recurring, persistent infections. Additionally, SCVs show increased resistance to antimicrobial treatment (Proctor et al. 2006). Most of the available research concerning SCVs and internalisation of staphylococci comes from human medicine. Staphylococci with confirmed SCV phenotypes include *S. aureus*, *S. epidermidis*, *S. lugdunensis*, *S. capitis*, and *S. pseudintermedius* (Proctor et al. 2006; Maali et al. 2016; Bogut and Magryś 2021). Only one report known to the authors mentions internalisation of any staphylococcus in poultry – the internalisation of *S. aureus* into the osteoblasts of chicken embryos after experimental subcutaneous inoculation at 17** **days of age (Reilly et al. 2000). The prevalence of SCVs in other staphylococcal species is under debate. It is also unknown if these forms occur naturally among staphylococci in poultry.

According to the literature, MSCRAMMs relevant for the development of bone and joint diseases include fibronectin binding proteins (FnBPs), collagen binding protein (Cna), and clumping factor (Clf). However, it is important to note that no single factor has been found to be specific to bone infections (Wright and Nair 2010). FnBP of *S. aureus* has two isoforms – FnBPA and FnBPB, with very similar domain organisations and sequences. Occurrence of both isoforms varies across the population (Josse et al. 2017). FnBPs have a high affinity to fibronectin, fibrinogen, and elastin. They play a crucial role in adhesion to the host cell and can trigger internalisation (Hauck and Ohlsen 2006). Cna binds to type I collagen and has demonstrated a crucial role in the development of experimental septic arthritis and osteomyelitis, and has been an important factor for haematogenous dissemination of *S. aureus* that lead to bone infection (Patti et al. 1994; Elasri et al. 2002; Xu et al. 2004). Two clumping factors – ClfA and ClfB – bind mainly to fibronectin, but they have also been found to bind to other matrix components, such as cytokeratin and loricrin (Foster 2019). Clf has been shown to contribute to endovascular infections (Moreillon et al. 1995; Entenza et al. 2000; Claes et al. 2017) and to play a crucial role in septic arthritis (Josefsson et al. 2001).

Staphylococci express a wide range of toxins and exoenzymes that interfere with host immune cells and can destroy host tissues (Gordon and Lowy 2008). The expression of such virulence factors is mainly controlled by global regulatory systems such as *agr*, *sarA*, and *sae* (Giraudo et al. 1997; Cheung et al. 2004). It has been shown that *S. aureus* clones lacking the *agr* or *sae* regulatory loci expressed a limited exoprotein profile. At the same time, *agr*/*sae*-lacking clones caused less pathogenic bone remodelling in mice and lowered intraosseous bacterial survival (Cassat et al. 2013).

Detailed analysis of *S. aureus* genomes allowed to discover a specific *S. aureus* that has infected chickens since the 1980s. Furthermore, this chicken-restricted clade has been undergoing continuous evolution which has resulted in acquisition of additional adhesins, new virulence determinants, and mobile genetic elements, such as pathogenicity islands (SaPIs). Genome analysis indicated that a particular SaPI may play a significant role in the BCO pathogenesis and virulence of this microorganism (Ekesi et al. 2021).

## 3. Bacterial chondronecrosis with osteomyelitis (BCO)

BCO was first reported in Australia in 1972 in turkeys. The most commonly isolated bacteria were *S. aureus*, followed by *E. coli* (Nairn 1973). Nowadays, it has become the most important cause of lameness in broilers in the world. In the veterinary literature, this disease has been described under such names as: femoral head necrosis (FHN), osteomyelitis, long bone necrosis, degeneration of the proximal femur, bacterial chondronecrosis (BCN) (McNamee and Smyth 2000). Presently, it is believed that the name BCO best reflects the nature of the changes observed macro- and microscopically, and at the same time it refers to the causative agents of the disease (bacteria). It is also not limited to a given localisation of lesions, as in the case of FHN (McNamee and Smyth 2000; Wideman 2016). BCO consists of bacterial infection and necrosis occurring primarily in the proximal ends of the femora and tibiotarsi. The bone ends can be divided into three regions: epiphysis, physis, and metaphysis. Other bones, especially fast-growing ones (including vertebrae), can also develop BCO lesions, although less frequently. BCO can occur unilaterally or bilaterally and in different locations in one bird. Most often, BCO occurs in chickens aged 14 to 70** **days, with the peak incidence around the day 35 (McNamee and Smyth 2000; Wideman 2016).

Bacterial infections are often mixed. *Staphylococcus aureus* is most commonly involved, although other staphylococci, *Enterococcus* spp., *E. coli*, or *Salmonella* spp. may be isolated (Emslie et al. 1983; Tate et al. 1993; Thorp et al. 1993; McNamee et al. 1999; Dinev 2009; Kolbjørnsen et al. 2011; Wideman 2016). Jiang et al. (2015) demonstrated in their study that there was a significant difference in the taxonomic diversity of bacteria from normal and BCO bone samples. BCO bone samples had less diversity in their bacterial communities, with overrepresentation of genera *Staphylococcus*, *Enterobacter*, *Serratia*, and *Nitrincola*.

Recent reports have revealed an increasing role of *S. agnetis*, coagulase-variable staphylococcal species, initially associated with dairy cattle mastitis. It was retrieved in high percentage from BCO lesions in broiler chickens in 2015 in the USA (Al-Rubaye et al. 2015). Further experimental studies proved that *S. agnetis* had an ability to induce lameness at a very high prevalence (cumulative lameness of over 80%) (Al-Rubaye et al. 2015; 2017). In 2017, during a longitudinal study of Danish broiler breeders, it was found that 2.7% of all deaths were associated with endocarditis and septicaemia caused by *S. agnetis*. This microbe was also isolated from the cloacae of newly hatched chicks originating from studied breeders which suggests that it is possible for *S. agnetis* to be transferred from the parent stock to the offspring (Poulsen et al. 2017).

The pathogenesis of BCO is not fully understood. Some possible causes of BCO development were named: the translocation of bacteria from damaged skin or mucous membranes to the bones through tissues or blood vessels, omphalitis, transovarian infections or infections through air sacs (McNamee and Smyth 2000). Regardless of the source of the bacteria, the presence of lesions in multiple locations in one bird almost certainly requires bacteraemia to occur. One hypothesis says that haematogenously spread opportunistic bacteria, that translocated across the epithelium of respiratory or digestive system, colonise damaged tissues near the epiphyses of the bones (Wideman 2016). It is suspected that disturbances in blood circulation within the proximal ends of bones and damage to epiphyseal and physeal cartilages play a key role in the development of BCO (Wideman et al. 2012; Wideman and Prisby 2012; Wideman 2016). The physeal cartilage is supplied with blood from two sides – from the side of the articular cartilage, and from the metaphysis. These blood vessels very rarely penetrate the entire thickness of the growth plate. Most often they form hair-loop ends, leaving a certain width of the growth plate non-vascularised. These vessels are characterised by a sluggish blood flow and the presence of large windows between endothelial cells which creates favourable conditions for circulating bacteria to colonise the cartilage near the physis (Howlett 1979; Howlett et al. 1984; Orth and Cook 1994; Wideman and Prisby 2012). The colonisation proceeds easier when microfractures are present in the cartilage, revealing its matrix. The growth plates of fast-growing species of poultry (meat-type chickens, turkeys) are extremely susceptible to this type of damage. Compared with mammals, avian growth plates of the proximal ends of the long bones of the pelvic limbs are thicker due to their chondrocytes being arranged in long columns. Additionally, if a microfracture cuts a blood vessel near the physis, it creates a focal ischemia and subsequently necrosis, further contributing to bacterial infection (Howlett 1979; Howlett et al. 1984; Orth and Cook 1994; Wideman and Prisby 2012). Immunosuppression, caused by infectious and non-infectious factors, is thought to predispose birds to BCO (Emslie et al. 1983; Andreasen et al. 1993; Thorp et al. 1993; McNamee et al. 1999; Huff et al. 2000). The stress reaction is related to the secretion of immunosuppressive glycosteroids. Administration of dexamethasone (a glycosteroid) to birds has been shown to increase the incidence of BCO (Huff et al. 2000; Wideman and Pevzner 2012).

One of the factors contributing to BCO is the rapid growth of broilers (Kestin et al. 2001; Wideman and Prisby 2012; Prisby et al. 2014; Wideman 2016). Genetic selection for the best possible muscle mass gains of meat type chickens means that their bones are not able to develop proportionally to the increasing body weight. The leg bones of chickens are less mineralised, more porous and prone to fractures or other injuries due to the constant excessive mechanical force exerted on them (Thorp and Waddington 1997; Wideman and Prisby 2012; Prisby et al. 2014). Also, any other conditions predisposing to limb bone damage, such as tibial dyschondroplasia (Wyers et al. 1991) or rickets (Thorp and Waddington 1997), may increase the risk of BCO. Bone lesions can be easily colonised by opportunistic bacteria (Bradshaw et al. 2002; Knowles et al. 2008).

**Figure 1.**
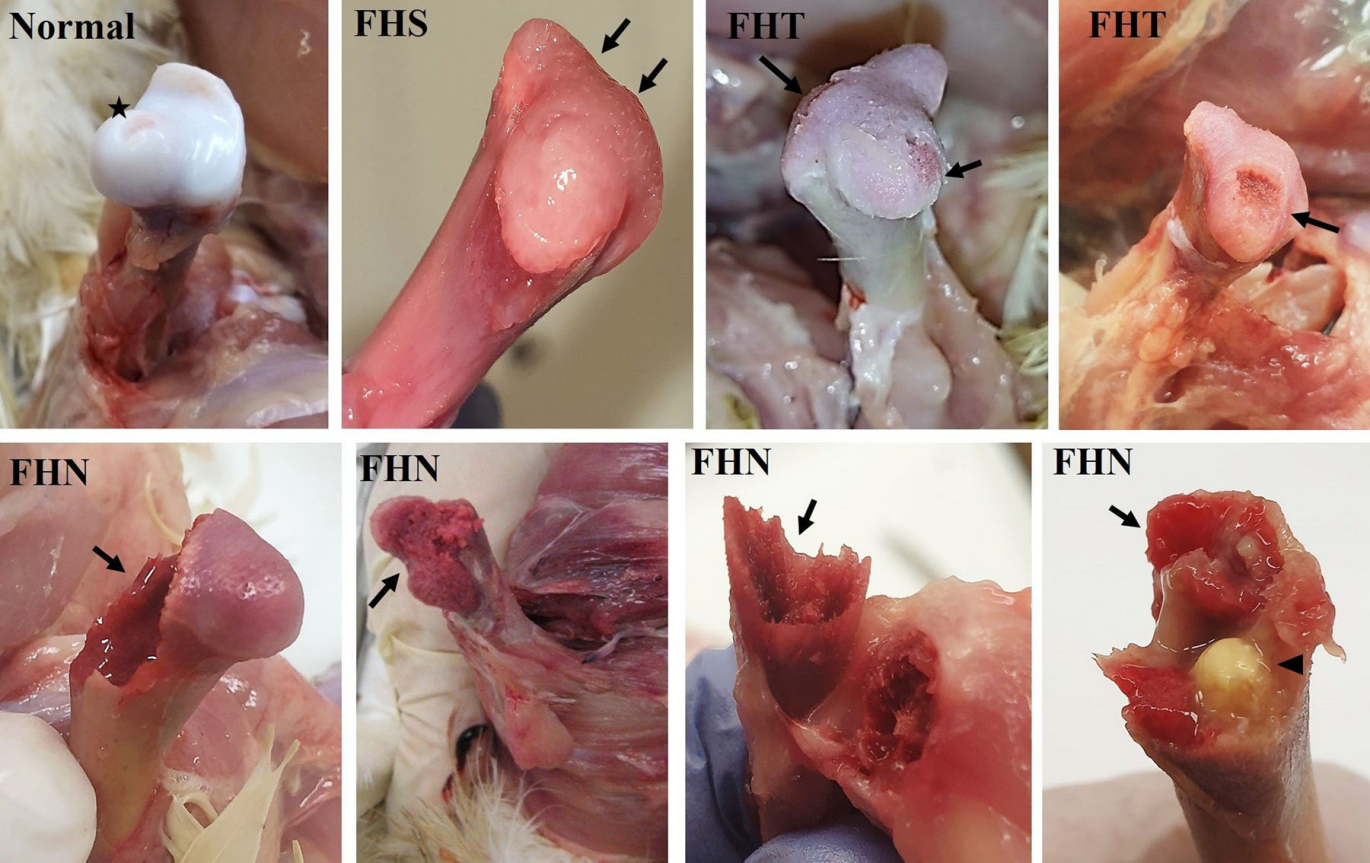
Stages of BCO lesions in proximal femoral head of broiler chicken. Normal head of femur, asterisk (★) shows the fovea for ligament of femoral head (fovea capitis femoris); FHS – femoral head separation (epiphyseolysis); FHT – femoral head transitional degeneration; FHN – femoral head necrosis; Arrow head (▶) indicates bacterial fibriscess. Arrows (→) indicate lesions. (Figure source: Authors).

BCO has a progressive nature. It most commonly originates on the border of the physis. The first stage is thought to be the femoral head separation (FHS), also known as epiphyseolysis. However, great care should be taken when disarticulating the femur to check for FHS. In some cases FHS can be attributed to the excessive use of force while disarticulating the femur from the acetabulum during necropsy, trauma caused by mishandling the bird or degenerative changes appearing when a long period of time has passed between death and necropsy (Wideman et al. 2012). Ligaments which fix the femoral head in the hip joint should be carefully cut with a scalpel blade during necropsy. When the hip joint was disarticulated correctly, even in apparently healthy birds, there were histopathological lesions consisting of clefts between epiphyseal and physeal cartilage that are believed to predispose to the development of BCO. This suggests that BCO does not develop from the clefts alone, but from the following bacterial infection (Wideman and Prisby 2012). Subsequently there may be progressive necrosis, ulceration, and fracture of the growth plate. Some authors refer to this stage as femoral head transitional degeneration (FHT) (Wideman et al. 2012). The final stage is femoral head necrosis (FHN), characterised by visible perforation, fractures, and necrosis along with osteomyelitis in the femoral head (Figure 1) (Butterworth 1999; Dinev 2009; Durairaj et al. 2009; Wideman et al. 2012). BCO lesions in the proximal end of tibiotarsus are usually referred to as tibial head necrosis (THN) (Butterworth 1999; Wideman and Prisby 2012).

## 4. Spondylitis

One of the forms of BCO in chickens is the fibronecrotic fibriscess formation in free thoracic vertebra (FTV, the sixth thoracic vertebra – Th6) or in vertebrae adjacent to it (Dolka and Szeleszczuk 2012; Wideman 2016). FTV is the only articulating element between notarium and synsacrum (Baumel et al. 1993). It is believed that mobility within FTV allows the mechanical forces to concentrate there, leading to microfractures in the cartilages (Carnaghan 1966; Wise 1971; Wideman 2016). The rarely reported spine fibriscess in locations other than FTV or its vicinity may develop because both notarium and synsacrum in young birds retain some mobility between the vertebrae forming them. This mobility allows for microfractures to appear in these sections of the spine. Similarly, as in long bones, BCO within spine forms when opportunistic bacteria are deposited via the blood flow in the areas of damage in vertebrae. This leads to progressive necrosis and fibriscess formation within the vertebral bodies. The vertebrae, enlarged by the fibriscess, begin to compress the spinal cord, leading to demyelination and necrosis of the nervous tissue. Clinical signs observed in such birds include lameness proportional to the degree of pressure on the spinal cord. Eventually, birds sit down with their legs stretched out in front of them, and a characteristic kyphosis (kinky back) may be visible (Wideman and Prisby 2012; Braga et al. 2016; Wideman 2016). In recent years, the most common bacteria isolated from spinal fibriscesses have been *Enterococcus* spp., in particular *Enterococcus cecorum* (Wood et al. 2002; Stalker et al. 2010; Dolka and Szeleszczuk 2012; Braga et al. 2016; Jung et al. 2018). However, single and mixed infections with *S. aureus* and *E. coli* have been reported (Wise 1971; Braga et al. 2016). In the study by Braga et al. (2016), *S. aureus* was identified in 14.3% of the vertebral osteomyelitis cases, being 7.1% in co-infection with *Enterococcus* spp. (*E. faecalis*, *E. hirae*), and not with *E. coli*. Carnaghan (1966) isolated *Staphylococcus pyogenes* (nowadays *S. aureus*) from spinal fibriscess (Th6–Th7) in broiler chickens and confirmed experimentally its ability to cause disease in 4–6-week-old chickens. It was shown that *S. pyogenes* can cause heterophilic fibriscess in the spine independent of its ability to produce haemolysin or coagulase. Similarly, Wise (1971) isolated *S. aureus* from lesions in the spine of a 6-week-old turkey and *Staphylococcus albus* (nowadays *S. epidermidis*) from a 6-week-old broiler. After the subsequent intravenous inoculation with *S. albus*, chickens developed spinal fibriscesses. The same lesions were found after inoculations with *S. aureus* strains (Kibenge et al. 1983; Griffiths et al. 1984).

## 5. Synovitis and arthritis

**Figure 2.**
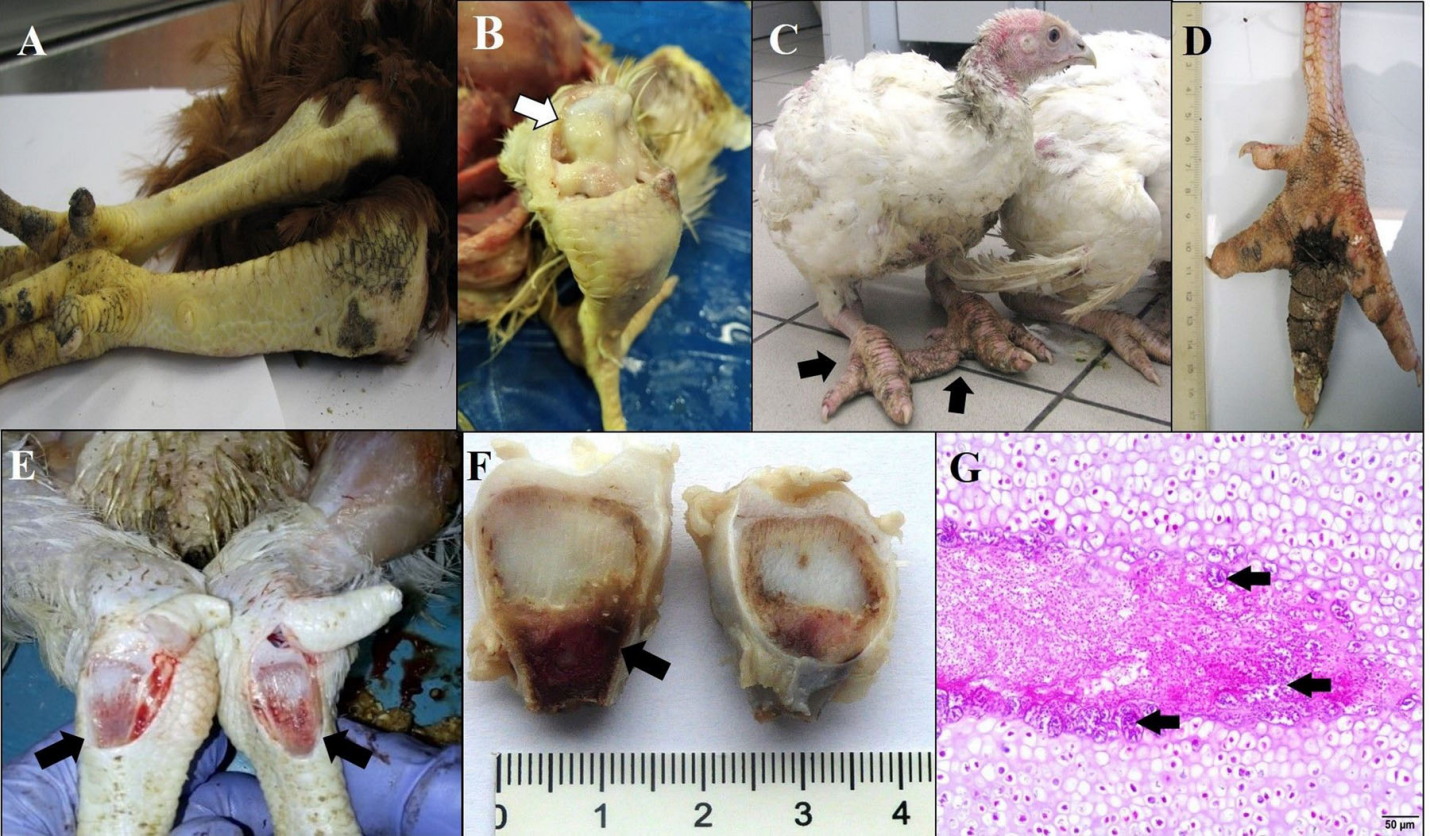
Examples of skeletal disorders associated with Staphylococcus infections in poultry A) Swollen hock joint and tarsometatarsus indicating unilateral arthritis, tenosynovitis in a 19-week-old Hy-Line Brown rooster. B) Arthritis of the left hock joint in a 5-week-old broiler chicken – swelling and yellowish fibrinous exudate (arrow). C, D) Bumblefoot in a 11-week-old turkey: swollen feet, toes (arrows) and characteristic black scab on footpad (beside a normal foot in another turkey). E, F, G) Dyschondroplasia with bacterial osteomyelitis (arrows) of tarsometatarsus in a broiler chicken. F) Sagittal section through the distal tarsometatarsus shows mass of cartilage and osteomyelitis. G) Necrosis of cartilage and multiple (intralesional) bacterial colonies (arrows). Haematoxylin-eosin. 100×. (Figure source of A–E: Authors; Source of F–G: Izabella Dolka, Department of Pathology and Veterinary Diagnostics, Institute of Veterinary Medicine, Warsaw University of Life Sciences – SGGW, Poland).

Synovitis is synonymous to active inflammatory arthritis. Synovitis caused by *Staphylococcus* spp. is one of the earliest described infections in poultry (Hinshaw and McNeil 1952; Sahu and Munro 1969). The most commonly affected joints are hock, metatarsal, and toe joints. Such joints are hot, painful, and swollen. Similar changes can be palpated along the tendons. During post-mortem examination, fibrinous or caseous exudate may be found in joints (Miner et al. 1968; Nairn and Watson 1972; Nairn 1973) (Figure 2A and B). Progressive lameness is observed in birds, they are reluctant to move, they prefer to sit on hocks or lay on breasts, their feathers are dirty and dishevelled. The prevalence of swollen hock joints in 6-week-old broiler flocks may range from 9 to 50%. Bacteria were recovered from 71% of hock joint samples, and *Staphylococcus* (60%) constituted the major genus among them (Awan and Matsumoto 1998). In another study, 30% of total *Staphylococcus* isolates (18/60) were recovered from joint samples of clinically sick birds (Wieliczko et al. 2002). Tsai et al. (2015) found bacteria in 57% (51/90) of the arthritic joints of Taiwan native coloured broiler chickens, and *Staphylococcus* accounted for 47% (24/51) of culture-positive cases (Tsai et al. 2015). Staphylococci can colonise the synovium causing local necrotic lesions, heterophile infiltration, and fibroblast proliferation (Miner et al. 1968). Joint and tendon sheath lesions are often accompanied by parallel changes in the bones (BCO). Joint infections appear to be secondary to bone infections (Nairn and Watson 1972; Alderson et al. 1986). Alderson et al. (1986) suggested that BCO and synovitis probably have a common pathogenesis and can be treated as a single disease complex.

## 6. Turkey osteomyelitis complex (TOC)

Formerly known as synovitis, osteomyelitis and green liver syndrome (SOG), the turkey osteomyelitis complex (TOC) is a syndrome caused by many opportunistic bacterial species, the most common of which are *E. coli* and *Staphylococcus* spp. (Droual et al. 1996; Huff et al. 2000). In a study by Bayyari et al. (1994), *S. aureus* and *E. coli* were the most frequently isolated species from bones, followed by *S. lentus*, *E. faecalis*, *Actinobacillus calco*, *Pseudomonas* sp. Some of the green livers contained Gram-positive cocci within the necrotic foci. Droual et al. (1996) isolated coagulase-negative staphylococci from joints and liver in different turkey flocks; however, *E. coli* was almost always isolated from bones, and from about 50% of joint and liver samples. *Salmonella* Heidelberg was isolated from one joint, and *Mycoplasma* spp. was not identified. TOC usually affects fast-growing tom turkeys older than 9–10** **weeks (Bayyari et al. 1994). Clinical signs include lameness and swollen joints. Turkeys may also show no clinical signs, and only in the slaughterhouse a green-coloured liver with necrotic foci would be found. However, in about 50% of cases, the green colour of the liver may be unrelated to TOC lesions, and these lesions may also occur separately to the green liver (Bayyari et al. 1994; Mutalib et al. 1996). TOC lesions include inflammatory lesions in bones and joints, and fibriscesses in soft tissues. The bone lesions are usually located at the proximal ends of tibiotarsi (Bayyari et al. 1994). Mutalib et al. (1996) in a study involving seven turkey flocks found that arthritis and osteomyelitis do not necessarily occur together in the same bird, and that they occur separately as often as together. Lesions in bones and joints could occur in many places in one bird, as well as individually. The most common locations of bone lesions were the proximal ends of the tibiotarsus (64.2%), humerus (24.2%), and femur (23.3%). Less frequently, the lesions were found in the distal end of the femur (3.3%) and, interestingly, in the ribs (10.8%). Joint lesions usually were found in the shoulder (62.6%), knee (36.6%), and hip (20.3%) joints.

It is believed that stress and immunosuppression play a key role in the development of TOC, as it has been seen in BCO (Wyers et al. 1991; Huff et al. 1999; 2000; 2001). It has been shown that even the stress associated with the daily handling and lifting of the turkeys at a young age contributes to a higher risk of TOC later in their lives (Huff et al. 2001). In another experiment, turkeys developed TOC after they were infected with pathogenic *E. coli* via air sacs with simultaneous administration of dexamethasone. Dexamethasone is a synthetic glycosteroid that mimic the body’s stress response and leads to immunosuppression. Interestingly, with subsequent doses of dexamethasone, *S. aureus* (although not administered) was more often isolated from the lesions both with and without *E. coli* challenge. It may suggest that immunosuppression is of greater importance for the development of TOC than the virulence of the bacteria (Huff et al. 1999; 2000).

## 7. Bumblefoot

Bumblefoot, known as ulcerative pododermatitis or footpad dermatitis, is a chronic inflammation of the metatarsal (plantar) and/or digital pads in poultry and captive wild birds (Martland 1984; Wilcox et al. 2009; Heidemann Olsen et al. 2018; Thøfner et al. 2019). Although bumblefoot is not a skeletal disorder *per se*, it is one of the most common conditions that can lead to lameness in poultry (Hester 1994). Foot pad dermatitis has a significant negative impact on broiler productivity. Jones et al. (2019) estimate an average incidence at 41% of broiler flocks in different poultry production systems in the UK, the Netherlands, and France. Reports of severe foot pad dermatitis vary depending on chicken rearing conditions, and they can be as low as average 2.8% (UK) (Dawkins et al. 2004) or as high as 70.8% (France) (Allain et al. 2009). Disease may enhance the rate of mortality by 12.7% compared with the normal mortality rate estimated for flock, and FCR may be reduced by 3.3% (1.06–4.35%) (Jones et al. 2019).

The disease has a complex aetiology that involves bacterial component and many predisposing factors associated with management, nutrition, genetics, sex, or body size (Martland 1984; Bilgili et al. 2009; Lay et al. 2011; Heidemann Olsen et al. 2018). *Staphylococcus aureus* was the most frequently isolated pathogen from bumblefoot cases (Satterfield and O’Rourke 1981; Hester 1994; Wilcox et al. 2009; Heidemann Olsen et al. 2018; Thøfner et al. 2019; Youssef et al. 2019). Heidemann Olsen et al. (2018) revealed a low genetic diversity among *S. aureus* isolates cultured from bumblefoot in layer hens, which may indicate a common source of infection. It seems that some *S. aureus* isolates may have specific characteristics facilitating them to cause infection in the foot pad tissue. Among other bacterial species were *S. hyicus, S. agnetis, S. lentus, S. simulans, E. coli, E. faecalis, E. hirae, Gallibacterium anatis, Proteus mirabilis, Pseudomonas aeruginosa, Trueperella pyogenes, Aerococcus urinaeequi* (Heidemann Olsen et al. 2018; Thøfner et al. 2019; Youssef et al. 2019).

Bumblefoot can arise from foot skin injuries serving as the portal of entry for bacteria and can lead to other conditions such as BCO or synovitis (Wilcox et al. 2009). Footpad can become extremely enlarged due to extensive swelling and deep ulcers (Figure 2C and D). The most frequent presentation of bumblefoot is a deep-seated plantar fibriscess covered by a thick black scab (Lay et al. 2011; Heidemann Olsen et al. 2018; Youssef et al. 2019; Andreasen 2020), previously described as ‘subdermal plantar abscessation’ (Butterworth 1999). Histopathologically, bumblefoot is characterised by chronic, proliferative, necrotising inflammation with a substantial number of Gram-positive cocci and complete destruction of the keratin and epidermal layer (Satterfield and O’Rourke 1981; Shepherd and Fairchild 2010; Heidemann Olsen et al. 2018). In more advanced stages, bacteria may invade deeper tissues, tendons, bones, or may enter the bloodstream leading to systemic infection (Wilcox et al. 2009; Shepherd and Fairchild 2010; Heidemann Olsen et al. 2018). Anyanwu et al. (2015) reported staphylococcosis in turkeys with a bumblefoot and a simultaneous occurrence of swollen head. *Staphylococcus aureus* was isolated from the fibrinous (viscid greyish-yellowish) exudate from foot lesions and head. Respiratory infection and septicaemia were excluded, the lesions arose from skin wounds (Anyanwu et al. 2015).

## 8. Dyschondroplasia with osteomyelitis

Dyschondroplasia is a skeletal disorder characterized by accumulation of abnormal cartilage masses in the epiphyseal growth plate of bones. Although it may affect the long bones (femur, humerus), tarsometatarsus or vertebrae in fast-growing meat-type chickens, turkeys, or ducks, the typical location is the proximal end of the tibiotarsus thus the name of diseases – tibial dyschondroplasia (TD). TD is described as multifactorial disease. The incidence of dyschondroplasia can be influenced by genetic, nutritional, environmental, and other non-infectious factors. Bacteria tend to be secondary to the induced lesions. Despite many studies, the mechanism of induction by different factors is still poorly understood (Thorp et al. 1993; Orth and Cook 1994; Julian 2005; Jahejo and Tian 2021). Due to the similar factors contributing to the disease, the association between TD and TOC incidences has been suggested (Huff et al. 2000).

The most frequent form of TD is subclinical stage. According to Julian (2005), 30–50% of male meat-type poultry may develop dyschondroplastic lesions without locomotor signs. Diagnosis occurs most often at an advanced, late stage of the disease, when birds show lameness, bone deformities associated with considerable pain and loss of body mass. Dyschondroplastic lesions may physically facilitate bacterial infection (Huff et al. 2000). Osteomyelitis and bone necrosis can constitute complications of dyschondroplasia (Wyers et al. 1991) (Figure 2E–G). The abnormal blood vessels that are unable to penetrate dyschondroplastic lesions may favour bacteria in the bloodstream to colonise and initiate inflammation. The most common cause of osteomyelitis is *S. aureus* which enters the bone by hematogenous spread (Wyers et al. 1991; Rath et al. 1994; Jiang et al. 2021). Other authors recovered *E. cecorum* from bilateral TD lesions in broiler chickens (Ekesi et al. 2021).

## 9. Amyloid arthropathy

Amyloid arthropathy is a form of AA amyloidosis (AAA) that occurs in chickens (Landman 1999). The disease can result from bacteraemia and is associated with the deposition of amyloid fibril protein within joints and various organs. Joint amyloidosis occurs from 5 to 6** **weeks of age onwards and causes chronic arthritis, lameness, and growth retardation (Landman 1999; Blanco et al. 2016). Amyloid arthropathy has been reported in brown layer breeds and rarely in broiler breeders. For unknown reasons, brown breeds are more susceptible to amyloid arthropathy than white breeds. The disease has not been reported in broiler chickens, probably due to their short production cycle that does not allow enough time for AAA to develop (Blanco et al. 2016).

The main aetiological agent associated with layer amyloid arthropathy is *Enterococcus faecalis*. Other bacterial species, i.e., *S. aureus*, *E. coli*, and *Salmonella* Enteritidis, have been reported to be able to induce amyloid arthropathy; however, the resulting lesions were much milder than those caused by *E. faecalis*. In contrast, in the cases of broiler breeder amyloid arthropathy, *S. aureus* was the most frequently isolated species (73.4%), followed by *E. faecalis* (25%) and *E. coli* (1.5%) (Landman et al. 1998).

## 10. Microbiome homeostasis and skeletal disorders in poultry

Recent studies have highlighted the importance of microbiome homeostasis in the development of skeletal disorders. Changes in the intestinal microbiota by antibiotics, diet or other factors may disturb the microbiota composition (dysbiosis), reduce the intestinal barrier function, and thus affect bone health. On the other hand, the modulation of the gut microbiota by the administration of various feed additives has been considered as an effective strategy for the prevention and treatment of skeletal disorders in poultry (Kogut 2013; Tong et al. 2018; Jiang et al. 2021).

Metagenomic studies revealed differences in gut microbiota between chickens with TD (tibial dyschondroplasia) and healthy chickens (Tong et al. 2018). TD-chickens had lower abundant microbiota and diversity of intestinal microbiota. The authors indicated that it is the small intestine, not large, that plays a more important role in immunity and metabolism. However, there was no significant difference in the small intestine contents between TD and healthy chickens at the level of phylum. The phylum *Firmicutes* (which includes staphylococci) predominated in both chicken groups. Interestingly, a comparison of the large intestine bacterial contents at the phylum level indicated the predominance of the phylum *Proteobacteria* in TD-chickens. Although differences at the genus level were noted in the intestinal contents between TD and healthy chickens, the genus *Staphylococcus* was not found. The presence of gut pathogens associated with immunity and inflammation was higher in the intestines of TD-chickens, which may affect the angiogenesis on tibial growth plates and contribute to TD (Tong et al. 2018).

A metagenomic analysis indicated the association of chicken blood microbiota with BCO pathogenesis. (Mandal et al. 2016). Furthermore, potential bacterial biomarkers in blood, associated with BCO, were identified. The bacterial communities in the blood of BCO chickens were found to be largely distinct from those of healthy chickens. The phylum *Firmicutes* was enriched in BCO chickens compared with healthy ones. In addition, the genus *Staphylococcus* was present in the blood microbiota of BCO chickens, which confirms the importance of this genus in BCO pathogenesis. It was argued that microbiota dysbiosis may lead to BCO in chicken (Mandal et al. 2016).

Jiang et al. (2015) conducted a molecular survey of femoral and tibial heads of healthy and lame chickens. It was shown that, even macroscopically, normal bone samples possess complex bacterial communities with the dominant phylum *Proteobacteria* (>90%), followed by the phyla *Firmicutes* and *Actinobacteria*. There were significant differences between the bacterial communities of macroscopically normal bones and of those with BCO lesions. The genera *Staphylococcus*, *Enterobacter*, and *Serratia* were overrepresented in samples with BCO lesions, which can point to their potential role in BCO pathogenesis.

## 11. Treatment and prevention

### 11.1. Antibiotic therapy

Therapeutic antimicrobial administrations have been found to reduce the incidence of BCO and other staphylococcal diseases (Wideman et al. 2015; Andreasen 2020). Wideman et al. (2015) used therapeutic doses of enrofloxacin, which significantly reduced the incidence of BCO-associated lameness in broilers reared on wire flooring (26.2% in the enrofloxacin group vs 36.9% in the control group). Because of osteomyelitis being a highly destructive process, it is important that antimicrobial therapy be initiated as quickly as possible. Most cases of acute osteomyelitis can be managed by antimicrobials. Nearly all antibiotics penetrate well into bone and the articular tissue (Dowling 2013; Thabit et al. 2019). However, when acute osteomyelitis progresses into its chronic phase, it is extremely challenging to treat. Chronic osteomyelitis is characterised by necrotic foci or sequestra in bones that are enveloped by pathologically changed avascular tissue, which prevents the penetration of most systemic antibiotics, rendering them ineffective (Dowling 2013). There is little data available about the antimicrobial treatment of osteomyelitis in poultry. By extrapolating recommendations for the treatment of osteomyelitis in humans and other domestic animals, several weeks (4–6) of antimicrobial therapy would be required for a full remission of infection (Fraimow 2009; Dowling 2013). Such time frame makes antimicrobial therapy hard to justify in current trends in poultry husbandry. On top of that, due to many strains of staphylococci manifesting resistance to a broad range of antimicrobial agents and the trend to increase antimicrobial resistance in bacterial populations, antimicrobial therapy is not suitable to be a long-term solution to the problem (McNamee and Smyth 2000). Antimicrobial therapy cannot undo the damage already done to the skeletal system, and soon after withdrawing the antibiotic, BCO is likely to reoccur (Wideman et al. 2015). To further complicate the matter, sick and lame birds are often depressed, not willing to drink and eat, or have difficulty reaching food and drinking water. This makes them less likely to acquire a full dose of the administered drug. However, antimicrobial treatment of bumblefoot (pododermatitis) often results in clinical improvement of affected birds, especially when combined with improvement to rearing conditions. Minocycline (Satterfield and O’Rourke 1981) and levofloxacin (Youssef et al. 2019) were successfully used to treat bumblefoot in birds.

### 11.2. Vaccination

There have been decades of research and many attempts at creating a vaccine against *S. aureus* both for humans and cattle, some with promising results. However, no such vaccine has been released to date (Chang et al. 2008; Middleton 2008; Proctor 2012; Fowler and Proctor 2014; Klaas and Zadoks 2018; Misra et al. 2018; Moscoso et al. 2018; Redi et al. 2018; Alabdullah et al. 2020; Miller et al. 2020). Such a vaccine could provide useful knowledge for the development of vaccines for use in poultry (McNamee and Smyth 2000). Smith (1954) tried a live subcutaneous immunisation of chickens with a coagulase-positive non-haemolytic *Staphylococcus* spp. strain with no effect.

Despite ongoing efforts over the past years, there are no licensed vaccines currently available for poultry against staphylococcosis (Kaul et al. 2001; El-Maghraby et al. 2020). Genetic diversity among *S. aureus* isolates, large number of virulence factors, and immune-evasion strategies have provided considerable challenges in the production of vaccines.

### 11.3. Antibiotic alternatives

The growing consumer pressure for antibiotic-free poultry meat, attitudes to animal welfare, and demand for broilers with robust immune response to a variety of pathogens, have impact on the global poultry farming. Nutrition has a significant effect on the proper development and function of the skeletal system in fast-growing poultry. According to the literature, dietary levels of 8 vitamins, 13 elements, and 6 amino acids, as well as protein and energy, may be directly involved in leg health in poultry (Edwards 2000). Feed additives, such as mineral supplements (inorganic and organic), probiotics, prebiotics, phytobiotics (botanicals), organic acids, essential oils, immunostimulants, amino acids, and enzymes, serve as antibiotic alternatives and help to improve health performance of chickens (Haque et al. 2020; Jiang et al. 2021).

Probiotics can be useful in preventing staphylococcal infections by limiting the amount of staphylococci translocating through the intestinal epithelium into the blood stream (Watkins and Miller 1983; Wideman et al. 2012; 2015). Probiotics can work through several modes of action, such as: changing the pH of the intestine lumen, competing for nutrients with pathogenic bacteria, producing bacteriocins, strengthening the intestinal epithelium tight junctions, colonising the epithelium and preventing the adhesion of pathogenic bacteria, binding pathogenic bacteria, altering the gut microbiota, or modulating the immune system (Saint-Cyr et al. 2016). The more damaged the epithelium, the easier it is for bacteria to translocate out of the gut (Wideman 2016). However, Rojas-Núñez et al. (2020) found no correlation between subclinical necrotic enteritis, induced by *Clostridium perfringens* and *Eimeria maxima*, and the incidence of BCO in chickens. This may indicate that in the case of BCO, the gastrointestinal tract is not the point of entry for bacteria or that the way of experimental infection lacked some key components that are present and crucial in naturally occurring infection. The experimental model used in the study needed some modifications to better reflect natural infection. Another possibility is that damaged epithelium is not necessary for the bacteria to translocate from the intestine, i.e., the bacteria may harbour virulence genes that facilitate the translocation. Ciurescu et al. (2020) reported that *Bacillus*-based probiotics may serve as a potential strategy for increasing skeletal health in broilers. Dietary supplementation of a probiotic *Bacillus subtilis* strain (ATCC 6051a) in chicken diet significantly improved the quality parameters of the tibia and had a bacteriostatic effect on cecal *Staphylococcus* spp. and *E. coli*.

Mineral supplements Ca, P (and Ca:P ratio), Cl, Zn, and Cu are essential to improve bone quality and reduce lameness incidences (Edwards 2000). Muszyński et al. (2018) revealed a positive effect of dietary Cu and phytase supplementation on the bone metabolism and the articular cartilage thickness. Tibiae from supplemented chickens had better mechanical strength and were subjected to lower mechanical stresses. More recently, Alrubaye et al. (2020) demonstrated that the mixture of organic trace minerals (Avila-ZMC) reduced lameness by 20% in broilers raised on wire flooring and by 25% in broilers raised on litter flooring with a bacterial (*S. agnetis*) challenge via drinking water. Furthermore, the mineral supplementation enhanced the bactericidal activity of phagocytes and the intestinal barrier integrity. The authors concluded that the complex of trace minerals could be efficacious in reducing BCO lameness.

### 11.4. Environmental and management factors

Besides nutrition, many environmental and management-related factors such as reduction of heat stress, light intensity, and flock density, have been considered as crucial strategy in maintaining skeletal health in chicken (Nääs et al. 2012). Because staphylococci are opportunistic pathogens, their ability to cause disease is as much determined by the bacterium as by the host. Any factors that decrease the immunity of the host favour the development of infection. Thus, it is imperative that birds be well protected against immunosuppressive factors such as stress, infectious bursal disease virus, or chicken infectious anaemia virus (Mutalib et al. 1983; McNamee et al. 1999; Wideman and Pevzner 2012). Any factors that create a portal of entry for the bacteria should be addressed as well. These include but are not limited to rough and sharp edges, wire floors, quality of the litter, bird pecking, and enteric pathogens (McNamee and Smyth 2000; Wideman 2016).

Hatcheries can play an important role in spreading staphylococcal infections. Newly hatched chicks with open navels and immature immune systems can be readily infected. It has been shown that *S. aureus* could be recovered in high rates from hatcheries (Thompson et al. 1980; McCullagh et al. 1998; Rodgers et al. 2003) and that the younger chicks were infected with *S. aureus* the more incidences of BCO were observed in their life (McNamee et al. 1999). Proper hatchery hygiene, disinfection and biosecurity measures may reduce the risk of staphylococcal infections (McNamee and Smyth 2000).

As BCO develops when excessive biomechanical forces affect the immature skeleton, management strategies that slow down the growth rate of chickens have been successful in reducing the prevalence of BCO and skeletal disorders (Riddell 1983; Robinson et al. 1992; Yu and Robinson 1992; Havenstein et al. 1994; Havenstein et al. 2003; Su et al. 1999; Kestin et al. 2001; Julian 2005; Knowles et al. 2008; Bentley et al. 2020). One of such promising strategy is restrictive feeding. Programmes that reduce quantitative feed intake in the early life of chickens tend to reduce lameness with little or no loss of body weight at slaughter age (Yu and Robinson 1992; Su et al. 1999). Qualitive diet restriction either by restricting protein or metabolizable energy can also have a positive effect on gait scores (Hulan et al. 1980; Venäläinen et al. 2006). Slower growth at an early age due to feed restriction allows more time for the skeletal system of the bird to mature. For the same reason, poultry farmers could consider selecting slow-growing broiler breeds. It has been demonstrated that broilers growing <50** **g per day experienced better welfare, had better gaits and lower incidences of pododermatitis or hock burns than their faster-growing counterparts (Dixon 2020; Rayner et al. 2020).

## Conclusions

Staphylococcal infections are a common occurrence in poultry. Among the infections they cause, those of the skeletal system are some of the most, if not the most important infections. Skeletal disorders are associated with significant welfare issues and economic losses in poultry production worldwide. Strong genetic selection has produced birds that gain a lot of muscle mass at a very early age. Their immature skeletal system is unable to cope with such biomechanical stress. It leads to trauma to the epiphyses, which creates favourable sites for haematogenously spread bacteria to colonise. It leads to BCO, which is the most common cause of lameness in poultry. BCO induces lesions in bones and joints, which usually require some level of immunosuppression caused by bacteria such as *Staphylococcus* spp. and *E. coli* and appear in fast-growing, young birds.

Staphylococcal infections are hard to manage. Staphylococci are ubiquitous in the poultry farm environment. They are opportunistic pathogens and manifest a broad antimicrobial resistance. This means that prevention or treatment should be concentrated on both the pathogen and its host. Traditional antimicrobial treatment is of little use and should be implemented carefully, so as not to increase antimicrobial resistance. Management practices that reduce the growth rate in the early life of the birds seem to be effective in reducing the incidence of lameness. Birds should be protected from excessive stress and immunosuppressive agents, as they seem to play an important role in many forms of staphylococcosis.

Because of multiple forms of infections caused by staphylococci, the *S. aureus*-vaccine efficacy against one disease may fail against another. Access to genome-based technologies has considerably advanced the identification of antigen candidates for future vaccine development in poultry. However, the evasion mechanisms that *S. aureus* utilizes to counteract bird immune responses still remain a challenge in vaccine trials.
